# Investigations of Oligonucleotide Usage Variance Within and Between Prokaryotes

**DOI:** 10.1371/journal.pcbi.1000057

**Published:** 2008-04-18

**Authors:** Jon Bohlin, Eystein Skjerve, David W. Ussery

**Affiliations:** 1 Norwegian School of Veterinary Science, Oslo, Norway; 2 Center for Biological Sequence Analysis, Department of Systems Biology, Technical University of Denmark, Lyngby, Denmark; Washington University, United States of America

## Abstract

Oligonucleotide usage in archaeal and bacterial genomes can be linked to a number of properties, including codon usage (trinucleotides), DNA base-stacking energy (dinucleotides), and DNA structural conformation (di- to tetranucleotides). We wanted to assess the statistical information potential of different DNA ‘word-sizes’ and explore how oligonucleotide frequencies differ in coding and non-coding regions. In addition, we used oligonucleotide frequencies to investigate DNA composition and how DNA sequence patterns change within and between prokaryotic organisms. Among the results found was that prokaryotic chromosomes can be described by hexanucleotide frequencies, suggesting that prokaryotic DNA is predominantly short range correlated, i.e., information in prokaryotic genomes is encoded in short oligonucleotides. Oligonucleotide usage varied more within AT-rich and host-associated genomes than in GC-rich and free-living genomes, and this variation was mainly located in non-coding regions. Bias (selectional pressure) in tetranucleotide usage correlated with GC content, and coding regions were more biased than non-coding regions. Non-coding regions were also found to be approximately 5.5% more AT-rich than coding regions, on average, in the 402 chromosomes examined. Pronounced DNA compositional differences were found both within and between AT-rich and GC-rich genomes. GC-rich genomes were more similar and biased in terms of tetranucleotide usage in non-coding regions than AT-rich genomes. The differences found between AT-rich and GC-rich genomes may possibly be attributed to lifestyle, since tetranucleotide usage within host-associated bacteria was, on average, more dissimilar and less biased than free-living archaea and bacteria.

## Author Summary

There are potentially many factors responsible for how archaeal and bacterial genomes are composed. Recent advances in DNA sequencing have made it possible to use computational and statistical methods to examine the interplay between evolution and genomic composition. We wished to see whether particular properties could be extracted that would provide clues on how prokaryotic DNA is composed. For instance, we wondered whether or not protein coding regions carried a greater information potential than non-coding regions, if there is a link between genome size and GC content, whether GC content is different in coding and non-coding regions, and possible associations between DNA composition and environment. Our results indicated that genomic nucleotide frequencies are a determinant of many DNA compositional properties, but also that other influences are at work. For instance, bacteria are known to frequently exchange DNA with the environment and other organisms. Acquired DNA can therefore have different compositional properties than host DNA, and since pathogenicity and antibiotic resistance in bacteria is often associated with foreign DNA, advancing the knowledge of DNA composition is of great importance.

## Introduction

Prokaryotic DNA can be considered as a long chain of overlapping oligonucleotides, and frequencies of differently sized oligonucleotides can reveal different properties and patterns of bacterial and archaeal genomes. On average, roughly 86% of prokaryotic DNA codes for proteins [1] and thus a considerable amount of information is held in trinucleotide (codon) frequencies. Additional information however, can be found by studying other oligonucleotide sizes. Dinucleotide distributions, or nearest neighbor frequencies, are used to calculate base-stacking energies [2], while DNA structural properties can be calculated using di- to tetranucleotide frequencies [2]–[5]. In addition, the structures of A, B and Z type DNA helices are largely determined by 11-, 10- and 12-mers, respectively [2],[6]. Another advantage of considering genomic DNA as a set of fixed-sized oligonucleotide frequencies is that bias and pattern preference, i.e. the randomness inherent or lack thereof, in the complete DNA sequence can be detected [3]. Alternatively, DNA patterns can be investigated by examining occurrences of individual nucleotides [7],[8].

Oligonucleotide frequencies are very much influenced by codon distributions which, in turn, are correlated with GC-content [9]. Since GC content is correlated with the environment of prokaryotes [9],[10], so are oligonucleotide distributions [11]. The distributions of oligonucleotide frequencies can reveal other properties as well. GC skews, i.e. increased cytosine compared with guanine content on the leading strand, can be used to determine DNA replication start and stop positions in bacteria [12]. However, many archaeal and slow replicating bacterial genomes do not have pronounced GC skews (or AT skews) on leading and lagging strands, but replication start and stop positions can be detected with increased precision by examining oligonucleotide frequency skews with progressively larger oligonucleotide sizes [13].

In addition to the properties described above, transcription and regulation sites are also coded by certain oligonucleotide patterns [14]. Such oligonucleotides are therefore thought to be severely under- or overrepresented compared with what is expected from mean genomic GC content or compared to oligonucleotide frequencies found in other closely related species [14].

The examples above illustrate some of the properties that can be extracted from DNA sequences by examining oligonucleotide usage variance. This motivated us to explore how oligonucleotide distributions change within and between prokaryotes in coding and non-coding regions, how biased oligonucleotide frequencies are, and whether any particular trends could be detected. In order to do this a series of statistical tests were performed on all sequenced bacterial and archaeal chromosomes (up to September 2006). We found that tetranucleotide frequencies carried considerable genomic information potential, and were therefore used in all statistical tests based on oligonucleotide usage. The statistical tests included oligonucleotide usage deviation from mean (OUD, a measure of oligonucleotide frequency variations in genomes), and oligonucleotide usage variance from expected (OUV, a measure of randomness or bias [3]). These tests were used to examine how tetranucleotide frequencies fluctuated within chromosomes (OUD), and how tetranucleotide distributions differed from random tetranucleotide frequencies calculated by mean genomic nucleotide frequencies (OUV). OUV measures GC content both globally and locally in chromosomes with a 40 kbp non-overlapping sliding window and was used to calculate expected tetranucleotide frequencies as well as GC content in coding and non-coding sequences for each chromosome tested. These tests were performed for all sequenced 402 prokaryotic chromosomes at the time, and their corresponding open reading frames. The first test, however, was concerned with statistical information potential in different oligonucleotide sizes using a different approach than [3].

## Results/Discussion

### Information Potential in Oligonucleotides

**Figure 1:**
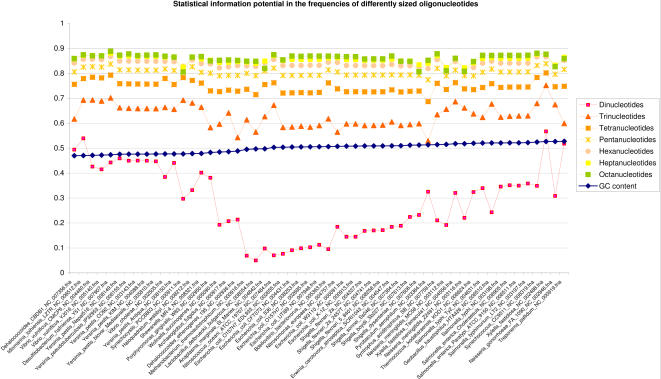
Statistical information potential in differently sized oligonucleotides. Cumulative information potential is measured in di- to octanucleotide frequencies in prokaryotic genomes with GC content between 47% and 53%. These genomes were selected because of the increased sensitivity of the Pearson correlation measure for chromosomes with similar AT/GC content. The archaeal and bacterial chromosomes are represented along the horizontal axis, sorted by increasing GC content from left to right, with corresponding correlation scores between observed *n*-mer words and approximated *n*-mer words on the vertical axis. The *n*-mer words were approximated by observed (*n*–*1*)-mer words and genomic nucleotide frequencies. High correlation scores indicate increased similarity between observed and approximated oligonucleotide usage.

We measured the statistical information carried by the differently sized oligonucleotides from di- to octanucleotides in prokaryotes with GC contents between 47% and 53%. From Figure 1, it can be observed that the largest increase in information was obtained by going from nucleotide frequency approximation of dinucleotides to trinucleotide usage approximations based on dinucleotide frequencies and GC content (details can be found in Materials and Methods). A more careful investigation of Figure 1 revealed that progressively less information was gained from usage approximations of tetranucleotides up to heptanucleotides, and practically no additional information appeared to be present in approximated octanucleotide frequencies. Thus, oligonucleotide sizes larger than hexanucleotides possess little additional information potential, if any, in prokaryotic DNA.

### GC Content in Coding and Non-Coding Regions

GC content was measured in coding and non-coding regions (see Materials and Methods for more detail) and it was found that non-coding regions were, on average, roughly ∼5.5% more AT rich (∼5.3% and ∼5.5%, for AT and GC rich chromosomes respectively) than coding regions, with the assumption that 14%, on average, of each chromosome was non-coding DNA [1].

Why non-coding regions are ∼5.5% more AT rich than coding regions may be related to the DNA curvature in promoter regions, and possibly termination sites [15], and to lower stacking energies found in AT rich DNA patterns compared with GC rich [2]. Increased AT content in non-coding regions suggest less energy is required to split the double helix for transcription [2].

### GC Content and Genome Size

**Figure 2:**
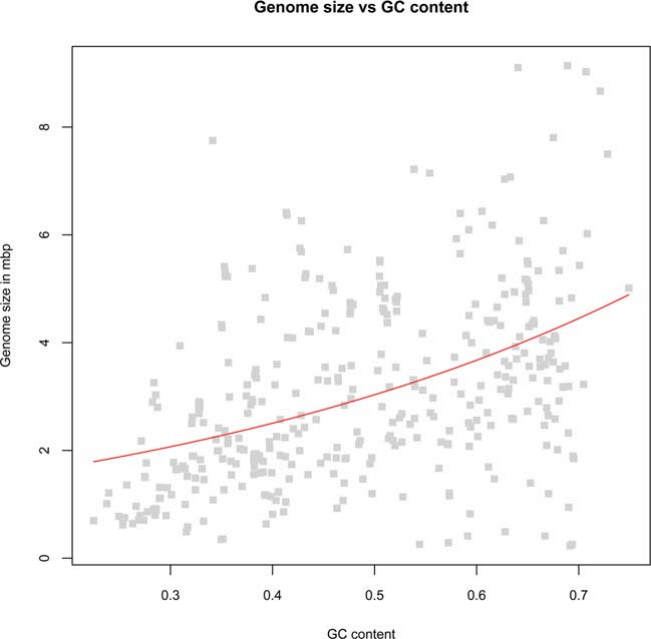
Prokaryotic genome size versus GC content. Prokaryotic chromosomes are sorted by increasing GC content from left to right on the horizontal axis. The vertical axis represents chromosome size in mbp.

Although the link between GC content and genome size has been debated [16],[17], we obtained significant (*P*<*0.001*) correlation with ρ = *0.47* (Spearman's rho) between chromosome size and GC content (see Figure 2). The following regression equation was fitted:
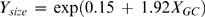
with the assumption of linear variance. *Y_size_* gives the size of the chromosomes (response) in mbp and *X_GC_* is global GC content (predictor, *P*<*0.001*).

### Bias in Tetranucleotide Usage

**Figure 3:**
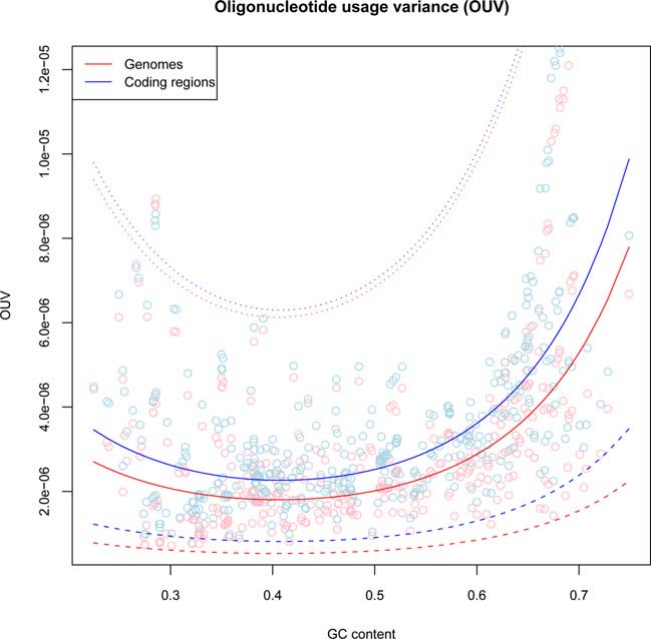
Tetranucleotide usage variance measures of 402 archaeal and bacterial chromosomes. Prokaryotic chromosomes are sorted by increasing GC content from left to right (vertical axis), with red and blue regression lines representing OUV values (horizontal axis) for chromosomes and coding regions, respectively. Larger values imply more bias, or stronger selectional pressure, in genomic tetranucleotide usage. The surrounding dotted lines indicate 99% prediction intervals.

We measured how tetranucleotide usage varied in genomes compared with expected tetranucleotide usage. This expected tetranucleotide usage was calculated from mean genomic GC content, and implicitly assumes that each nucleotide in every tetranucleotide, and therefore also the whole chromosome, is independent of its neighbors. In other words, the more similar observed and expected tetranucleotide frequencies are, the more random (i.e. less biased) are the observed tetranucleotide frequencies, and thus the genomic DNA composition. Figure 3 shows how OUV varied between genomes compared to genomic GC content. Significant correlation was found between GC content and OUV values using the following regression equation:
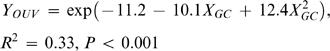

*Y_OUV_* designates genomic OUV values (response) while the predictor, *X_GC_*, represents GC content. Our results showed that GC rich archaea and bacteria tended to have a less random DNA composition than AT rich. The reason for this is not known, but it has been argued [3] that thermodynamic properties of tetranucleotides may be important, i.e. base stacking energy and curvature. Tetranucleotide usage variance in coding regions was found to be even more strongly correlated with global GC content:
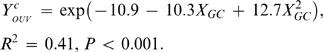

*Y^C^_OUV_* designates OUV values in coding regions (response), while *X_GC_* is global GC content (predictor).

Thus, bias in tetranucleotide usage in non-coding regions was less affected by global GC content than coding regions.

Preliminary tests on a set of sequenced genomes involving randomization by increasing AT/GC content similarly to non-coding % size produced significantly larger differences in tetranucleotide usage bias than what was observed for non-coding regions. This indicates that non-coding regions do carry information and are exposed to selectional pressures and bias although considerably less than coding regions. This can be seen from Figure 3 where bias in tetranucleotide usage in coding regions increased more with GC content than tetranucleotide usage in non-coding regions. It should be noted that the above analysis is based on average values from concatenated DNA sequences, and nothing is stated about how OUV values vary within chromosomes and coding regions. Preliminary tests indicate that OUV values vary considerably within archaeal and bacterial chromosomes.

### Tetranucleotide Usage Variation Within Genomes

OUD gives a measure of how homogeneous or heterogeneous genomes are in terms of DNA composition. The OUD (but not OUV) measure can also detect to what extent tetranucleotide patterns are distributed throughout the genome. Low OUD values thus indicate increased similarity and not increased randomness. In contrast to OUV, the OUD measure is calculated as the average variance of oligonucleotide occurrences within the chromosome based on oligonucleotide frequencies from a non-overlapping 40 kbp sliding window compared with mean genomic oligonucleotide frequencies.

**Figure 4:**
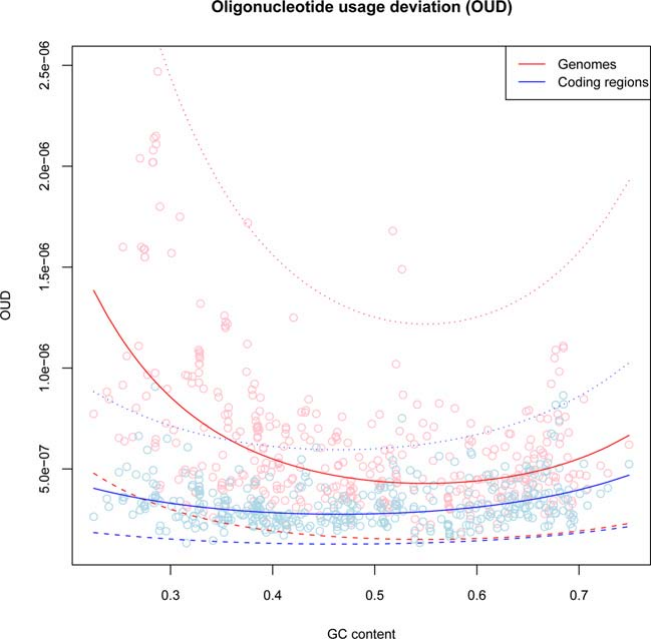
Average oligonucleotide usage deviance within prokaryotic chromosomes (OUD). Each archaeal and bacterial chromosome is sorted on the horizontal axis with increasing GC content from left to right, with corresponding OUD values on the vertical axis. The red and blue lines represent OUD scores for whole chromosomes and coding regions, respectively. Smaller OUD values mean more homogeneous chromosomes in terms of tetranucleotide usage. The surrounding dotted lines indicate 99% prediction intervals.

From Figure 4 it can be observed that OUD scores were lower in coding regions (blue line) than in chromosomes containing both coding and non-coding regions (red line). Genomic OUD scores were progressively decreasing with increasing GC content indicating that tetranucleotide patterns in non-coding regions become progressively more similar with growing GC content. The following regression equations were obtained:
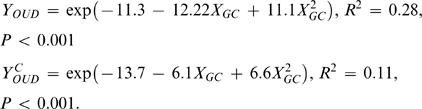
The response-functions *Y_OUD_* and *Y^C^_OUD_* represent OUD scores in genomes and coding regions, respectively, while the predictor *X_GC_* is GC content.

**Figure 5:**
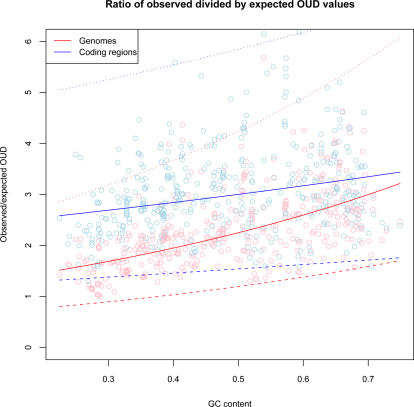
Ratio of observed divided by expected OUD values. The vertical axis shows the ratio of observed divided by expected OUD values for each chromosome sorted with respect to genomic GC content from left to right on the horizontal axis. The ratio test measures how observed oligonucleotide usage varies within chromosomes (red line) and coding regions (blue line) compared with expected based on GC content. Rising ratio values above 1 (vertical axis) means increased observed variance compared with expected. The dotted lines represent 99% prediction intervals.

Difference in tetranucleotide usage within genomes was supported by a ratio test where observed tetranucleotide usage variance within genomes was divided by expected tetranucleotide variance approximated by nucleotide frequencies. From Figure 5 it can be observed that considerably less variation was detected in the coding regions (blue line) compared with chromosomes containing both coding and non-coding regions (red line) with the following regression equations:
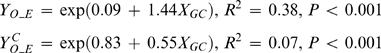

*Y_O_E_* and *Y^C^_O_E_* (response) are the ratios of observed divided by expected OUD values for genomes and coding regions respectively, while *X_GC_* represents mean global GC content (predictor).

This variance is, however, connected to larger fluctuations in GC content within genomes in AT rich prokaryotes. See the below section on variance in GC content within genomes for more detail. No correlation was found between OUD values and genome size.

### Variation of GC Content Within Genomes

**Figure 6:**
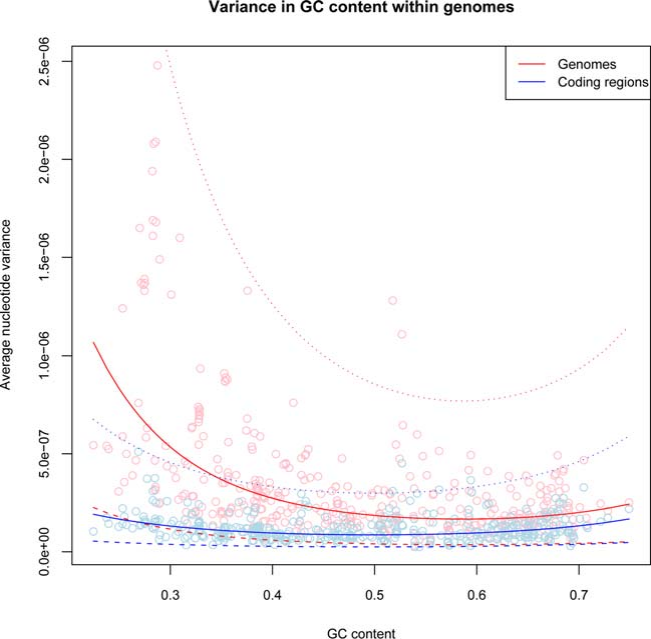
Variation of GC content within genomes. The vertical axis shows the variance of nucleotide frequencies within chromosomes (red line) and coding regions (blue line) compared with corresponding mean genomic GC content on the horizontal axis. Lower average nucleotide variance scores (vertical axis) means more similar distributions of GC content within chromosomes (and vice versa).

Predicted tetranucleotide usage based on genomic nucleotide frequencies was used to estimate variance in GC content within genomes (Figure 6). Since intrinsic tetranucleotide usage variance predictions were only based on nucleotide frequencies, these values were directly associated with fluctuations in local GC content obtained by comparing 40 kbp sliding windows with global (mean) GC content. We therefore wanted to investigate if fluctuations of intrinsic GC content showed any relation to global GC content, and whether there was a difference between coding and non-coding sections. Using regression analysis, significant correlation was found between global GC content and expected tetranucleotide usage variance, with the following equation:
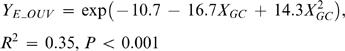

*Y_E_OUV_* (response) represents expected OUV usage and *X_GC_* GC content (predictor).

This result showed that there was significant correlation between global GC content and how GC content varied locally within genomes. The obtained correlation was also higher than what was observed for the OUD measure (*R^2^* = *0.28*), which means that variance in tetranucleotide frequencies within genomes is less sensitive to mean genomic GC content than variance in nucleotide frequencies.

Restricting the test to coding sections, correlation was found between global GC content and expected tetranucleotide usage variance, using the following equation:
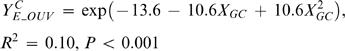

*Y^C^_E_OUV_* represents expected OUV values in concatenated coding regions (response), and the predictor, *X_GC_* , global GC content.

Thus, only weak correlation was found between fluctuations in intrinsic nucleotide frequencies and global GC content in coding regions. Since roughly 86% of prokaryotic DNA codes for proteins, on average, [1] the above result suggest that the GC content of non-coding regions within prokaryotes vary considerably, and that the rate of this variation within genomes is negatively correlated with mean genomic GC content. Hence, our results show that, on average, the more GC rich a genome is, the less variation of nucleotide frequencies are found in the non-coding regions, while the opposite is true for AT rich genomes, i.e. the more AT rich an archaeon or bacterium is the more varied are the nucleotide frequencies of the non-coding sections.

### Evolutionary Implications

Although a link was established between genomic GC content and OUV, (*R^2^* = *0.33*, see above section) AT rich bacteria with high OUV values and GC rich bacteria with low OUV values were also observed. High and low scores mean here being ranked among the 100 highest or lowest OUV scoring chromosomes of the 402 tested, respectively. In general, a larger number of AT rich bacteria were found with high variance scores than GC rich bacteria with low OUV scores. Closely related bacteria were also found at the opposite ends of the OUV score list. For instance, we found that “*Candidatus* Blochmannia floridanus” obtained an OUV value five times that of *Buchnera aphidicola* subsp. SG (ranked #28 and #356, respectively). Additional details can be found in Dataset S1. Both species are thought to have undergone genome reduction as they adopt a symbiotic lifestyle and have small genome size of comparable GC content [18]. Since low OUV scores imply high genomic mutation rates, different mechanisms may be responsible for the different tetranucleotide distributions in these genomes, with *Bl. floridanus* having a more mutated genome than *B. aphidicola*. Thus, different evolutionary mechanisms may still result in similar lifestyles. Both *Bl. floridanus* and *B. aphidicola* obtained similarly high OUD scores (respectively ranked #387 and #346), which means that they both rank among the prokaryotes with the most heterogeneous chromosomes. Host associated (including pathogenic) bacteria, in general, had the most heterogeneous genomes, while free living bacteria were the most homogeneous (see Dataset S1). It should also be added that no correlation was found between OUV values and genome size, thereby removing any link between random genome composition and genome size.

### Summary

Considering prokaryotic genomes from an oligonucleotide perspective, there seemed to be little increase in information potential in oligonucleotide sizes larger than hexanucleotides.

Comparing observed to expected tetranucleotide usage (OUV), we found that coding regions are, in general, more biased than non-coding regions, and more homogeneous according to the OUD test. GC rich genomes were also found to have more biased tetranucleotide frequencies than AT rich genomes. Although AT content increased in non-coding regions in both AT rich and GC rich genomes, it did not appear to be a consequence of tetranucleotide preference. OUD was found to decrease with increasing mean genomic GC content, indicating that GC rich genomes have a more homogeneous DNA composition, especially in non-coding regions. This result was additionally supported by a ratio test based on observed and expected OUD scores indicating that coding regions varied similarly for AT and GC rich genomes alike, while non-coding regions varied more within AT rich genomes. No correlation was found between OUV and OUD scores, implying that bias in tetranucleotide usage is not connected to intra-chromosomal homogeneity in prokaryotes.

## Materials and Methods

All sequenced archaea and bacteria available up to September 2006, (402 chromosomes and corresponding open reading frames, from 366 genomes in total) were downloaded from NCBI Genbank [19]. The different statistical tests were carried out with computer programs made according to the procedures described below. The free statistical package R [20] was used for visualization of results, regression analysis and curve-fitting. All analyses of DNA sequences were carried out in the 5′→3′ direction. Analysis of genomic coding regions was performed by concatenating every open reading frame for each chromosome into one large DNA sequence.

### Notation and Formulas

For notation we let *F_z_*(.) designate frequency with respect to a DNA sequence *z* of an independent variable consisting of a nucleotide (AT-content, *F_z_*(*w*), *w* is any nucleotide or *F_z_*(*A*), *F_z_*(*G*), *F_z_*(*C*) and *F_z_*(*T*)) or an oligonucleotide (*F_z_*(*w_1_w_2_…w_n_*), where *w_1_w_2_…w_n_* are nucleotides making up a DNA word consisting of *n* nucleotides. Correlation between frequencies of DNA words was performed with the standard Pearson correlation formula:
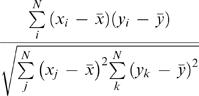
(1)
*x_i_* = *F^i^_x_*(*w_1_w_2_…w_n_*) represents DNA word frequency for word *i* and *x̅* is the average frequency for all possible combinations of DNA words consisting of *n* nucleotides (see below). The sums are taken for all possible oligonucleotide combinations, i.e. *N* = *4^n^*

Average values are found using the equation:
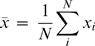
(2)


### Methods

The statistical information potential of the different oligonucleotide sizes was estimated by comparing frequency functions *F_x_*(*w_1_…w_n_*) to *F_x_*(*w_1_…w_n–1_*)*F_x_*(*w_n_*) using Formula 1 for genomes with AT content between 47% and 53%. Genomes with AT content in that range were chosen due to the increased sensitivity of the Pearson correlation method for chromosomes with similar AT/GC content. By leaving one nucleotide “free” the cumulative information in the different oligonucleotide sizes, i.e. combined information of all smaller oligonucleotides, could be measured.

GC-content in non-coding regions was calculated by finding GC-content in open reading frames and whole chromosomes with the following formula:
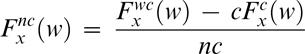
(3)where *c* = coding fraction measured by the sum of open reading frame sizes divided by corresponding chromosome size. Non-coding fraction is then *nc* = *1*–*c*.

The superscripts *c*, *nc*, *wc* designate GC content in coding, non-coding and whole chromosomes, respectively.

GLM based regression analysis was used to examine the relationship between global GC content (predictor) and genome size (response). Because of the highly non-linear nature of the data, Spearman's rank based correlation test was additionally used between genome size and global GC content.

OUV was calculated for each genome with the following formula:
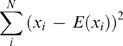
(4)where *E* is expected frequency for DNA word *x_i_* based on nucleotide frequencies:
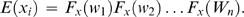
(5)The expected value is thus calculated based on the assumption that each oligonucleotide frequency consists of individual nucleotide frequencies independent of each other. Since Formula 5 bases calculations of the expected value only on genomic nucleotide frequencies it can be considered as a measure of how “random” the genomic composition of the organism is. Low OUV scores can therefore be interpreted to mean that a high degree of non-directed mutations has taken place within the genome, or, alternatively, that the individual nucleotides in each tetranucleotide are more ‘loosely’ tied to each other. High OUV scores may also be taken to mean that a larger degree of bias, or order, is present in the genomic oligonucleotide usage. An additional point of view is that OUV is a measure of information potential, where low OUV values mean that the DNA sequence in question has lower statistical potential of carrying information (and vice versa).

OUV was calculated for both whole chromosomes and coding regions. Regression analysis was then carried out between OUV values (response), both coding and whole chromosomes, and global GC content (predictor). The resulting regression equations, with corresponding coefficient of determination, here denoted by *R^2^*, can be found in the Results/Discussion section together with the corresponding *P* values.

The OUD test gives an average estimate of how oligonucleotide frequencies vary within prokaryotic chromosomes. Variance is calculated between the oligonucleotide frequencies calculated from a 40 kbp non-overlapping sliding window and mean genomic oligonucleotide frequencies with the following formula:
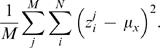
(6)Summations are taken over all possible oligonucleotide combinations *1*≤*i*≤*N*, and all non-overlapping sliding windows *1*≤*j*≤*M* in DNA sequence *x*. *z^j^_i_* = *F^i^_zj_*(*w_1_w_2_…w_n_*) represents the *n*-word frequencies for sliding window *j*, while μ*_x_* = *F_x_*(*w_1_…w_n_*) is the mean frequency of word *w_1_…w_n_* in DNA sequence *x*.

Regression analysis was performed for both whole chromosomes and coding regions with OUD as response and global GC content as predictor. The resulting regression equations with corresponding coefficient of determination, *R^2^*, and *P*-values can be found in the Results/Discussion section.

The ratio of observed divided by expected OUD values was additionally used to test whether any fluctuations in tetranucleotide frequencies could be detected in coding and non-coding regions. To calculate this, the OUD values obtained for each chromosome with Formula 6 was divided by the following equation:
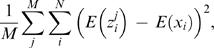
(7)which resulted in the formula:
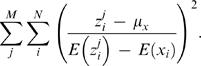
(8)In this case, the variance was calculated, for each chromosome, between expected oligonucleotide frequencies from a 40 kbp sliding window and expected oligonucleotide frequencies based on Formula 5.

Variation of nucleotide frequencies within genomes was calculated for each chromosome similarly to the OUD test, but expected oligonucleotide frequencies based on Formula 5 were used instead of observed. This is the same as calculating the variance between local and global GC content (i.e. GC content) and Formula 7 was used for this as well.

Regression analysis was performed on values obtained with Formula 7 and global GC content for both coding and whole chromosomes. The resulting equations, with the corresponding coefficients of determination, *R^2^*, and significance, can be found in the Results/Discussion section.

## Supporting Information

Dataset S1 Microsoft Excel file consisting of the data used to generate the results in the manuscript. Each column is labeled according to the abbreviations used in the text and additionally explained on a separate sheet.
(0.17 MB DOC)
