# Correction: Investigations of Oligonucleotide Usage Variance Within and Between Prokaryotes

**DOI:** 10.1371/annotation/4eb76620-9599-40f5-bb05-54f252e48f3b

**Published:** 2009-07-10

**Authors:** Jon Bohlin, Eystein Skjerve, David W. Ussery

In the Introduction, 2nd paragraph, the 4th sentence is incorrect. It should read: "GC skews, i.e. increased guanine compared with cytosine content on the leading strand, can be used to determine DNA replication start and stop positions in bacteria [12]."

