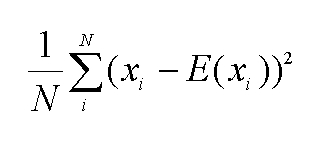# Correction: Investigations of Oligonucleotide Usage Variance Within and Between Prokaryotes

**DOI:** 10.1371/annotation/3785bc7c-4548-4554-8331-74cc68b8f356

**Published:** 2009-07-10

**Authors:** Jon Bohlin, Eystein Skjerve, David W. Ussery

Equation 4 is incorrect. See the correct equation here: