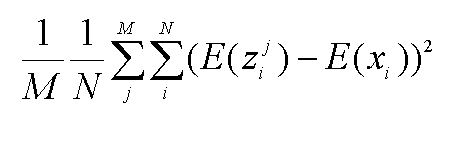# Correction: Investigations of Oligonucleotide Usage Variance Within and Between Prokaryotes

**DOI:** 10.1371/annotation/27a4f12b-e6e6-4d19-bf53-09ce05dfe1b9

**Published:** 2009-07-10

**Authors:** Jon Bohlin, Eystein Skjerve, David W. Ussery

Equation 7 is incorrect. See the correct equation here: