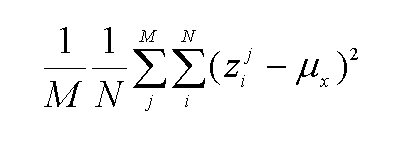# Correction: Investigations of Oligonucleotide Usage Variance Within and Between Prokaryotes

**DOI:** 10.1371/annotation/91dc9016-fc1e-495e-8828-22608c3efe44

**Published:** 2009-07-10

**Authors:** Jon Bohlin, Eystein Skjerve, David W. Ussery

Equation 6 is incorrect. See the correct equation here: