# Correction: Investigations of Oligonucleotide Usage Variance Within and Between Prokaryotes

**DOI:** 10.1371/annotation/45b4b21a-d0d1-40d2-8efd-c30b28baf1eb

**Published:** 2009-07-10

**Authors:** Jon Bohlin, Eystein Skjerve, David W. Ussery

Throughout the paper it was written that the test E_OUV gives a measure of how GC content varies within genomes. This is not accurate, since the formula (E_OUV) is concerned with all nucleotides (A, G, C, and T). A more fitting description is "Variation of base composition within genomes." Thus, the equation:

YE_OUV=exp(-10.7-16.7XGC+14.3X2GC),

actually describes how base composition, measured as varying 4-tuples of mononucleotide frequencies, varies within genomes. 

